# PROLACTIN-Deficiency in Adult Offspring of Diabetic Mothers

**DOI:** 10.1155/EDR.2000.31

**Published:** 2000

**Authors:** Leona Aerts, Rieta Van Bree, F. André Van Assche

**Affiliations:** 1 Department Obstetrics and Gynecology U.Z. Gasthuisberg K.U. Leuven. Herestraat 49 Leuven B-3001 Belgium

## Abstract

Maternal diabetes induces fetal alterations, resulting in lasting consequences for the glucose tolerance of the offspring over several generations. In our experimental rat model, circulating prolactin, oestradiol, progesterone and corticosterone levels, known to influence insulin secretion and action, are determined in plasma of female adult offspring of mildly and severely diabetic mothers. Prolactin and progesterone levels are equally low in both groups as compared to controls, stressing the involvement of the CNS in the transgeneration effect; oestradiol and corticosterone levels are normal. No correlation is found between these hormonal alterations and the known differences in glucose tolerance.


*Int. Jnl. Experimental Diab. Res.*, Vol. 1, pp. 31–38
Reprints available directly from the publisher
Photocopying permitted by license only

© 2000 OPA (Overseas Publishers Association) N.V.
Published by license under
the Harwood Academic Publishers imprint,
part of The Gordon and Breach Publishing Group.
Printed in Malaysia.

### PROLACTIN-deficiency in Adult Offspring of Diabetic Mothers

LEONA AERTS*, RIETA VAN BREE and F. ANDRÉ VAN ASSCHE

*Department Obstetrics and Gynecology, U.Z. Gasthuisberg, K.U. Leuven. Herestraat 49, B-3001 Leuven, Belgium*

*(Received 4 June 1999; In final form 23 August 1999)*

**Maternal diabetes induces fetal alterations, resulting in lasting consequences for the glucose tolerance of the offspring over several generations. In our experimental rat model, circulating prolactin, oestradiol, progesterone and corticosterone levels, known to influence insulin secretion and action, are determined in plasma of female adult offspring of mildly and severely diabetic mothers. Prolactin and progesterone levels are equally low in both groups as compared to controls, stressing the involvement of the CNS in the transgeneration effect; oestradiol and corticosterone levels are normal. No correlation is found between these hormonal alterations and the known differences in glucose tolerance.**

*Keywords:* Prolactin, progesterone, oestradiol, corticosterone, offspring of diabetic mothers

### INTRODUCTION

Diabetes of the mother during pregnancy induces alterations in the developing fetus, resulting in lasting consequences for the glucose tolerance in the offspring, even over several generations (Aerts *et al.*, 1990). Stimulated insulin response thereby is impaired throughout life and also insulin uptake (Aerts *et al.*, 1988) and action (Holemans *et al.*, 1991a) can be disturbed in these adult offspring. In the youngsters of mildly diabetic mothers islet- and B-cell mass are normal, but stimulated insulin response is deficient; in the youngsters of severely diabetic mothers islet- and B-cell mass are hyperplastic, insulin response is excessive and animals are insulin resistant (Aerts *et al.*, 1988; Holemans *et al.*, 1991a; Aerts *et al.*, 1997).

In fact, with regard to insulin, the adult youngsters of severely diabetic mothers in many aspects display features of pregnant rats. Also, during *normal pregnancy*, there is a doubling of the amount of endocrine pancreas and of B-cells (Aerts *et al.*, 1997), an increased recruitment of the number of B-cells (Aerts and Van Assche, 1998) and clear hyperactivity of the B-cells already in basal conditions (Aerts and Van Assche, 1975). Basal insulin levels are increased, the threshold for stimulated insulin secretion is decreased and the amount of secreted insulin above threshold is increased (Parsons *et al.*, 1992). The sensitivity of the insulin-sensitive tissues decreases during gestation, leading to severe physiological insulin resistance (Leturque

*Corresponding author. Tel.: 32/16/34.61.92, Fax: 32/16/34.42.05, e-mail: Leona.Aerts@uz.kuleuven.ac.be

*et al.*, 1984). In the *offspring of severely diabetic mothers*, all these features are present already in the non-pregnant animal, and they do not aggravate during gestation (Aerts *et al.*, 1988; Holemans *et al.*, 1991a; Aerts *et al.*, 1997; Aerts and Van Assche, 1998; Holemans *et al.*, 1991b). Therefore, also in these non-pregnant animals, those factors should be evaluated that are considered responsible for the pregnancy-related adaptations in the endocrine pancreas and the insulin-sensitive tissues.

Placental lactogen is considered to be an inducing factor for the islet adaptations during pregnancy (Parsons *et al.*, 1992). In non-pregnant animals its effect can be mimicked by hypophyseal prolactin (Brelje and Sorenson, 1991). The high estrogen levels of pregnancy, especially in combination with high progesterone concentrations, and in the presence of adequate corticosterone levels, contribute to an enhanced activity of the pancreatic B-cells (Faure *et al.*, 1983; Sutter-Dub *et al.*, 1978; Aerts *et al.*, 1980). Increased progesterone levels are involved in the development of gestational insulin resistance (Leturque *et al.*, 1989).

The aim of the present study is to evaluate the involvement of these hormones in the induction of islet- and B-cell hyperplasia, increased B-cell activity and insulin resistance in the adult non-pregnant youngsters of diabetic mothers.

### MATERIALS AND METHODS

The experimental animals were adult (age 80 to 94 days) female youngsters of pregnant Wistar rats, made mildly (MD) or severely (SD) diabetic by a single intravenous injection of Streptozotocin (Upjohn, Belgium) on the first day of gestation. They were compared to control (CO) female Wistar rats of the same age. Maternal glycemia levels in the different experimental groups were similar to those described in previous publications (Aerts *et al.*, 1990): mean values for CO 120, MD 160, SD 450 mg% at day 6 of gestation. Animals were kept in standard laboratory conditions; only litters with 8 to 12 pups were included in the study, pups were fed by their own mothers and weaned at the age of 20 days. From the age of 80 days, the oestric cycle of the female youngsters was followed daily for two weeks, by examining the vaginal smear. In this period, a small blood sample was taken from the tip of the tail of each animal at the four different stages of their cycle. Prior to this manipulation, the rats were put in an oven at 30°C for 15 minutes, in order to avoid excessive stress and to facilitate the blood sampling. After these two weeks, as the cycle of each rat was well established, the animals in preoestrus were anaesthetized with pentobarbitone (Nembutal) and allowed to accommodate for 30 minutes; then blood was taken from the aorta. Venous and arterial blood samples were collected in the morning from fed animals. The guidelines approved by the local institution for the care and use of animals were followed.

On the small samples of venous blood, prolactin levels were determined with a RIA for hypophyseal rat prolactin (Amersham). On the larger samples of arterial blood, plasma concentrations were determined for oestradiol 2 (Immuno 1, Bayer), progesterone (Axsym, Abbott), corticosterone (RIA), insulin (RIA) and glucose (glucose oxidase method, Beckmann). Data are expressed as Mean $\pm$ Standard Deviation (number of animals) and are statistically compared with a two-tailed two-sample student t-test for non-equal variances.

### RESULTS

The **oestric cycle** of the rats, with a periodicity of four days, is divided in four stages, recognizable on basis of the histology of the vaginal smears. Pro-oestrus (P) is characterized by the presence of nucleated epithelial cells, oestrus and the immediate post-oestric period (E) by masses of cornified cells, and the postovulation days (C3

and C4) by the presence of leucocytes. The oestric cycles of female MD and SD youngsters are similar to those of control rats.

Determination of basal **prolactin** levels is complicated by the sensitivity of the hormone to stress and to anesthesia. Therefore venous blood was taken from the tip of the tail of conscious animals, without anesthesia but after a period of accommodation to avoid excessive stress. Prolactin levels are significantly lower in the MD and the SD youngsters than in CO rats, when overall data of each group are compared (which is appropriate, since each stage of the oestrus cycle is represented in a similar way for each animal) (Fig. 1). The large standard deviations within each group are due to differences in prolactin levels between individuals throughout the cycle. A clear cyclic effect, however, is evident in each animal and in each group, with clear differences between the experimental groups. When considering the four stages of the cycle separately (Fig. 2), the main difference is clearly situated in the immediate post-oestrus stage (E), where values for MD and SD offspring are significantly below CO levels, the difference from control values being similar in both groups. Values remain low in these youngsters in the whole postovulatory period (C3 and C4) but are no longer significantly different from control levels.

**Oestradiol and progesterone** concentrations, for technical reasons (large samples of clean blood are needed for routine analysis), are determined on blood samples taken from the aorta at the end of the experimental period. Therefore each of these animals can be represented in only one stage of its cycle. The immediate pre-oestrus period (P) was chosen as reference period, since from preliminary studies on a limited number of rats, it was clear that the differences were most pronounced at that stage. Oestradiol levels follow a normal oestric cycle in each group (unpublished observations) without differences between the CO, MD and SD offspring, as demonstrated at stage P (Fig. 3). In this pre-oestric period, progesterone levels are

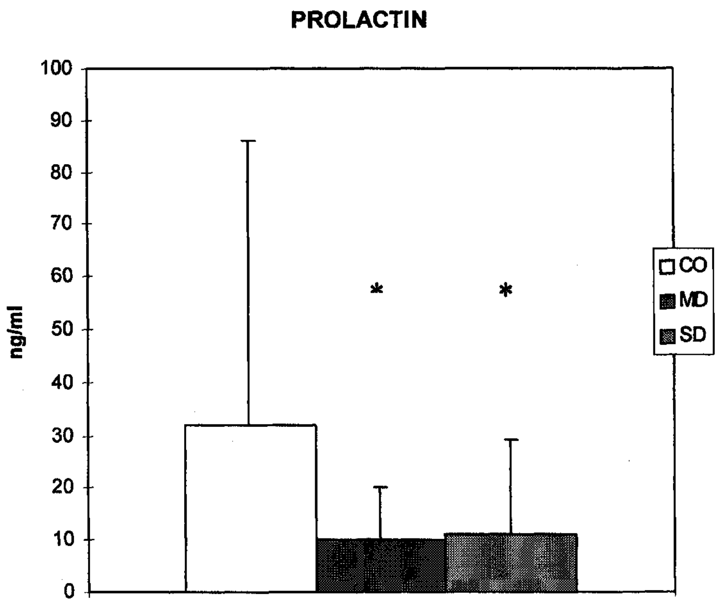

FIGURE 1 Prolactin levels in plasma (venous blood without anaesthesia) from adult female offspring of control (CO), mildly diabetic (MD) and severely diabetic (SD) mothers, regardless the oestric cycle of the animals. Data are expressed as Mean ± S.D., number of samples is 40, 36 and 36 respectively; differences from the CO group are considered significant (*) if $P < 0.05$.

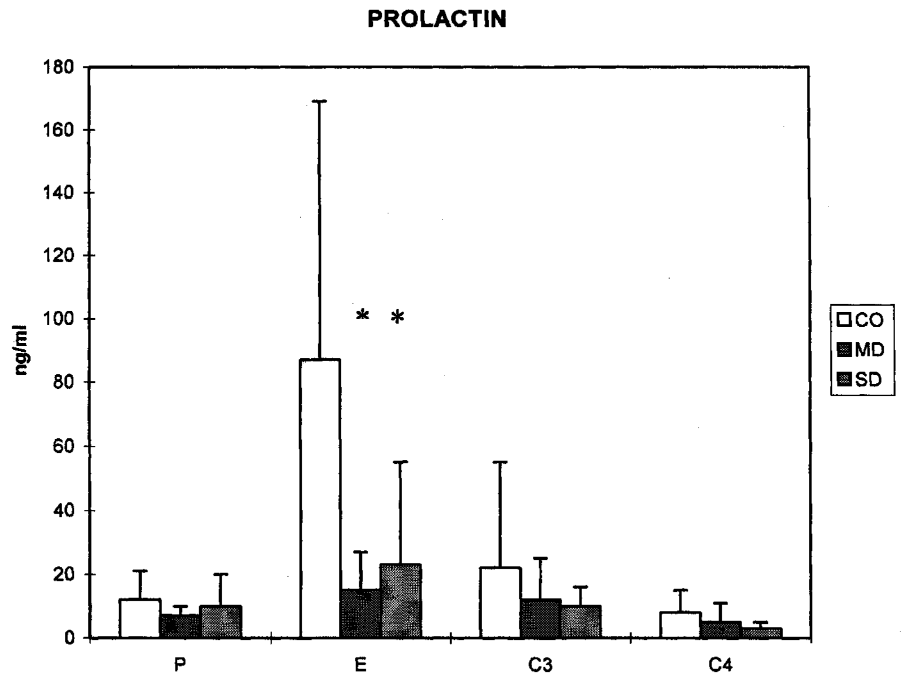

FIGURE 2 Prolactin levels in plasma (venous blood without anaesthesia) from adult female offspring of control (CO), mildly diabetic (MD) and severely diabetic (SD) mothers, at pre-oestrus (P), in the immediate post-oestrus period (E), and at day 3 and 4 of the cycle (C3, C4). Data are expressed as Mean ± S.D., number of animals is 10, 9 and 9 respectively; differences from the CO group are considered significant (*) if $P < 0.05$.

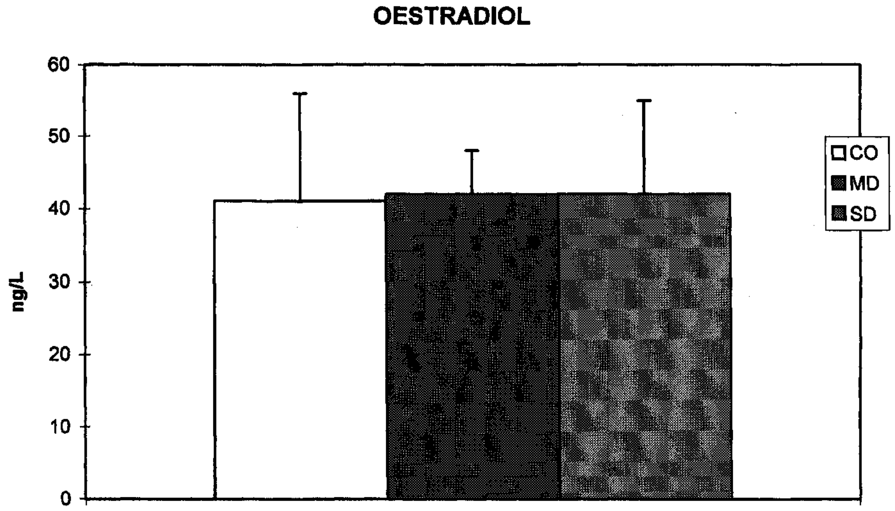

FIGURE 3 Oestradiol-2 levels in plasma (arterial blood taken under anaesthesia) from adult female offspring of control (CO), mildly diabetic (MD) and severely diabetic (SD) mothers, in the pre-oestric (P) stage of the cycle. Data are expressed as Mean ± S.D., number of samples is 10, 9 and 9 respectively; differences from the CO group are considered significant (*) if $P < 0.05$.

significantly lower in the MD and SD offspring than in the CO rats; the difference from the control value is similar in both groups (Fig. 4). This tendency is already seen in the late post-ovulatory period (C4), but disappears at oestrus (E, C3) (unpublished observations).

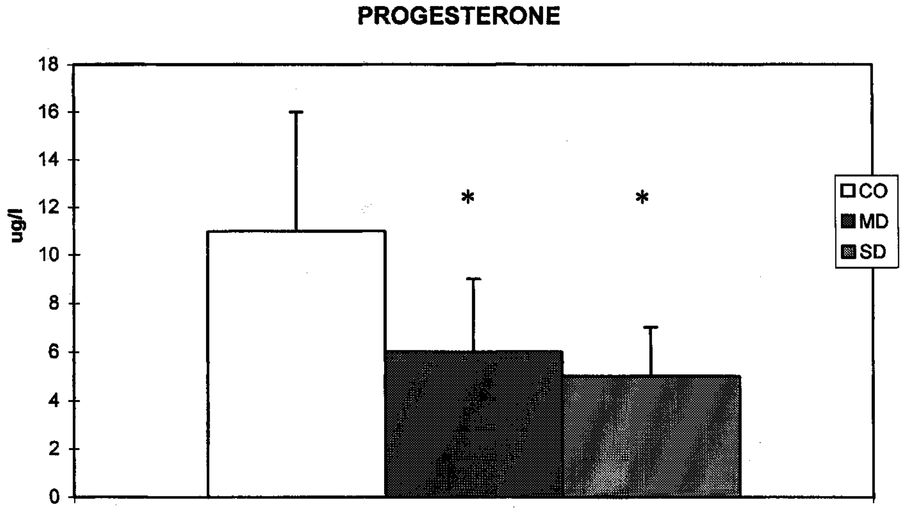

FIGURE 4 Progesterone levels in plasma (arterial blood taken under anaesthesia) from adult female offspring of control (CO), mildly diabetic (MD) and severely diabetic (SD) mothers, in the pre-oestric (P) stage of the cycle. Data are expressed as Mean ± S.D., number of samples is 10, 9 and 9 respectively; differences from the CO group are considered significant (*) if $P < 0.05$.

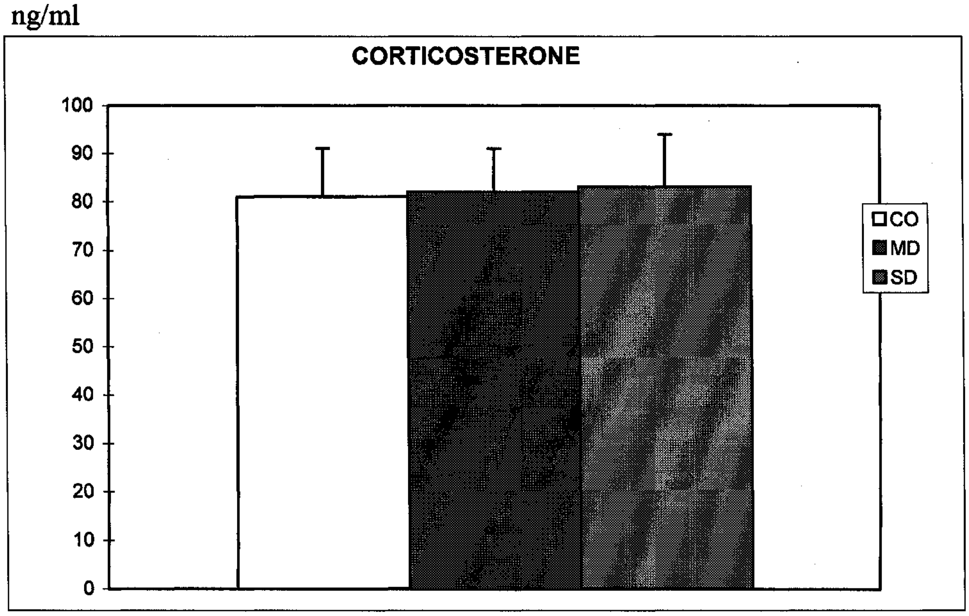

FIGURE 5 Corticosterone levels in plasma (arterial blood taken under anaesthesia) from adult female offspring of control (CO), mildly diabetic (MD) and severely diabetic (SD) mothers, in the pre-oestric (P) stage of the cycle. Data are expressed as Mean ± S.D., number of samples is 10, 9 and 9 respectively; differences from the CO group are considered significant (*) if $P < 0.05$.

TABLE I Glucose and insulin concentrations in plasma from arterial blood taken under anaesthesia from fed adult female offspring of control (CO), mildly diabetic (MD) and severely diabetic (SD) mothers. Data are expressed as Mean ± S.D., number of samples in parentheses; differences with the CO group are considered significant (*) if $P < 0.05$

| | CO | MD | SD |
|---|---|---|---|
| Glucose (mg/100 ml) | 181 ± 21 (10) | 195 ± 23 (9) | 175 ± 11 (9) |
| Insulin (uU/ml) | 52 ± 32 (10) | 99 ± 42 (9)* | 106 ± 10 (9)* |

**Corticosterone** levels are similar in the three groups of animals (Fig. 5). Basal **glucose** levels are not different between the three groups; the values are high since the blood samples are taken from fed animals after a period of anesthesia.

**Insulin** concentrations are significantly higher than normal in both experimental groups, confirming previous results on animals under the influence of anesthesia (Aerts *et al.*, 1990), (Tab. I).

### DISCUSSION

The adaptation of the endocrine pancreas during normal pregnancy is considered to be induced by placental lactogen (Brelje *et al.*, 1993). The increase of its levels during pregnancy indeed parallels the extension of the endocrine pancreas and the enhanced activity of its B-cells (Parsons *et al.*, 1992). Moreover, when islets from neonatal or adult rats are cultured in the presence of prolactin, islet volume, mitogenic activity and insulin secretion are drastically increased, while addition of growth hormone has little or no effect (Brelje and Sorenson, 1991). Dwarf mice lacking hypophyseal prolactin have a very reduced islet mass, while transgenic mice expressing human growth hormone (which has a lactogenic activity in mice) have an increased B-cell mass (Parsons *et al.*, 1995). The results of these experiments point to the lactogenic rather than the somatotropic hormones as inducers of the B-cell hyperplasia and hyperactivity during pregnancy.

Since B-cell hyperplasia and hyperactivity are also features of non-pregnant offspring of severely diabetic mothers, determination of prolactin levels in these animals appeared appropriate. However, prolactin is decreased instead of increased in these animals, as well as in the MD youngsters with a normal endocrine pancreas. The difference from control values is highly significant in both groups, despite the large standard deviations, which are due to the differences in prolactin levels between individuals. A relation between hypophyseal prolactin and islet expansion in these non-pregnant animals therefore should be excluded.

Also, oestradiol and progesterone are involved in the altered glucose homeostasis of pregnancy. Progesteronemia parallels insulinemia and oestradiol levels increase at the end of gestation. Moreover, administration of progesterone and oestradiol up to pregnant values to non-pregnant castrated female rats increases circulating insulin levels up to high values as seen in pregnancy, at least if adequate corticosterone concentrations are present (Faure *et al.*, 1983; Sutter-Dub *et al.*, 1978). Oestradiol directly increases insulin synthesis and secretion by the B-cells, the effect being more pronounced in combination with progesterone (Sutter-Dub *et al.*, 1978; Aerts *et al.*, 1980). Administration of progesterone for several days increases the replication rate in the islets of Langerhans of pregnant and non-pregnant rats (Nieuwenhuizen *et al.*, 1998), and induces insulin resistance (Leturque *et al.*, 1989). In our experimental groups, however, oestradiol and corticosterone levels were completely normal, and progesterone levels were decreased instead of increased, and to the same level in both groups. These low progesterone concentrations in both MD- and SD-offspring certainly do not correlate with the normal and hyperplastic islet mass found respectively in these animals.

From the present data it is clear that the altered glucose metabolism in the offspring of diabetic mothers is not directly related to those hormones that are responsible for similar adaptations during pregnancy. First, there is no correlation between the concentrations of these hormones and the characteristics of the endocrine pancreas. Secondly, the changes in hormone levels are remarkably similar in both groups, despite their difference in glucose homeostasis.

The significance of the low prolactin and progesterone levels is far from clear yet. Nevertheless, it is interesting to note that the presence of these hormones is also altered in the adult offspring of diabetic mothers, and to the same extent in both experimental groups, despite the marked differences between MD and SD youngsters for numerous other parameters (Aerts *et al.*, 1990). The origin and the consequences of the low prolactin levels in these animals, especially, need further attention. The deleterious effect of fetal hyperglycemia and hyperinsulinemia on the development of the fetal hypothalamus, resulting in later glucose intolerance (Plagemann *et al.*, 1992), includes alterations of the hypothalamic catecholamines. Indeed, dopamine and norepinephrine are increased in specific nuclei of the hypothalamus of neonatal and weaning pups of gestationally diabetic rats (Plagemann *et al.*, 1998). The high levels of dopamine, which is a prolactin-inhibiting factor, might be responsible for the persisting low prolactin levels in these animals, affecting thereby the secretion of progesterone from the corpus luteum.

It can be concluded that, while oestradiol and corticosterone levels are normal, circulating prolactin and progesterone levels are decreased in a similar degree in adult non-pregnant offspring of mildly and severely diabetic mothers. These alterations are not related to the adaptations in the endocrine pancreas of these animals. They might originate from effects of fetal hyperglycemia and hyperinsulinemia on the developing hypophyseal-hypothalamic system. The data add support to the concept of a neuroendocrine involvement in the transmission of a disturbed glucose tolerance to the offspring of diabetic mothers.

### *References*

Aerts, L. and Van Assche, F. A. (1975) Ultrastructural changes of the endocrine pancreas in pregnant rats. *Diabetologia*, **11**, 285–289.

Aerts, L. and Van Assche, F. A. (1998) Ultrastructural evaluation of B-cell recruitment in virgin and pregnant offspring of diabetic mothers. *Diab. Res. and Clin. Pract.*, **41**, 9–14.

Aerts, L., Van Assche, F. A., Faure, A. and Sutter-Dub, M. T. (1980) Effects of treatment with progesterone and oestradiol-17 beta on the endocrine pancreas in ovariectomized rats: ultrastructural variations in the B-cells. *J. Endocrinol.*, **84**, 317–320.

Aerts, L., Sodoyez-Goffaux, F., Sodoyez, J. C., Malaisse, W. J. and Van Assche, F. A. (1988) The diabetic intrauterine milieu has a long-lasting effect on insulin secretion by B-cells and on insulin uptake by target tissues. *Am. J. Obstet. Gynecol.*, **159**, 1287–1292.

Aerts, L., Holemans, K. and Van Assche, F. A. (1990) Maternal diabetes during pregnancy: consequences for the offspring. *Diabetes Metab. Rev.*, **6**, 147–167.

Aerts, L., Vercruysse, L. and Van Assche, F. A. (1997) Morphometric evaluation of the islets of Langerhans in an experimental model of impaired glucose tolerance and gestational diabetes. *Diab. Res. and Clin. Pract.*, **38**, 9–19.

Brelje, T. L. and Sorenson, R. L. (1991) Role of prolactin *versus* growth hormone on islet B-cell proliferation *in vitro*: implications for pregnancy. *Endocrinology*, **128**, 45–57.

Brelje, T. C., Scharp, D. W., Lacy, P. E., Ogren, L., Talamantes, F., Robertson, M., Friesen, H. G. and Sorenson, R. L. (1993) Effect of homologous placental lactogens, prolactins, and growth hormones on islet B-cell division and insulin secretion in rat, mouse, and human islets: implication for placental lactogen regulation of islet function during pregnancy. *Endocrinology*, **132**, 879–887.

Faure, A., Sutter-Dub, M. T., Sutter, B. C. J. and Assan, R. (1983) Ovarian-adrenal interactions in regulation of endocrine pancreatic function in the rat. *Diabetologia*, **24**, 122–12.

Holemans, K., Aerts, L. and Van Assche, F. A. (1991a) Evidence for an insulin resistance in the adult offspring of pregnant Streptozotocin-diabetic rats. *Diabetologia*, **34**, 81–85.

Holemans, K., Aerts, L. and Van Assche, F. A. (1991b) Absence of pregnancy-induced alterations in tissue insulin sensitivity in the offspring of diabetic rats. *J. Endocrinol.*, **131**, 387–393.

Leturque, A., Burnol, F. A., Ferre, P. and Girard, J. (1984) Pregnancy-induced insulin resistance in the rat: assessment by glucose clamp technique. *Am. J. Physiol.*, **246**, E25–31.

Leturque, A., Haugel, S., Sutter-Dub, M. T., Maulard, P. and Girard, J. (1989) Effects of placental lactogen and progesterone on insulin stimulated glucose metabolism in rat muscles *in vitro*. *Diabet. Metab.*, **15**, 176–181.

Nieuwenhuizen, A. G., Schuiling, G. A., Bisschop, L. G., Hilbrands, M., Moes, H. and Koiter, T. R. (1998) Proliferation of pancreatic islet cells in cyclic and pregnant rats after treatment with progesterone. *Horm. and Metab. Res.*, **30**, 649–655.

Parsons, J. A., Brelje, T. C. and Sorensen, R. L. (1992) Adaptations of islets of Langerhans to pregnancy: increased islet proliferation and insulin secretion correlates with the onset of placental lactogen secretion. *Endocrinology*, **130**, 1459–1466.

Parsons, J. A., Bartke, A. and Sorenson, R. L. (1995) Number and size of islets of Langerhans in pregnant, human growth hormone-expressing transgenic, and pituitary dwarf mice: effect of lactogenic hormones. *Endocrinology*, **136**, 2013–2021.

Plagemann, A., Heidrich, I., Rohde, W., Gotz, F. and Dorner, G. (1992) Hyperinsulism during differentiation of the hypothalamus is a diabetogenic and obesity risk factor in rats. *Neuroendocrinol Lett.*, **5**, 373–378.

Plagemann, A., Harder, T., Lindner, R., Melchior, K., Rake, F., Rittel, F., Rohde, W. and Dorner, G. (1998) Alterations of hypothalamic catecholamines in the newborn offspring of gestational diabetic mother rats. *Developmental Brain Research*, **109**, 201–209.

Sutter-Dub, M. T., Faure, A., Aerts, L. and Van Assche, F. A. (1978) Effects of progesterone and 17-beta-oestradiol treatments on the pancreatic B-cell castrated female rats. Biochemical variations. *J. Physiol.* (*Paris*), **74**, 725–730.

